# Caregiver Perceptions Regarding Alternative Emergency Medical Services Dispositions for Children: A Cross-Sectional Survey Analysis

**DOI:** 10.5811/westjem.2022.5.55470

**Published:** 2022-07-02

**Authors:** Caleb E. Ward, Jonathan Gougelet, Ryan Pearman, Gia M. Badolato, Joelle N. Simpson

**Affiliations:** * Division of Emergency Medicine, Children’s National Hospital, Washington, District of Columbia; † The George Washington University School of Medicine & Health Sciences, Washington, District of Columbia

## Abstract

**Introduction:**

Emergency medical services (EMS) systems have developed alternative disposition processes for patients (including leaving the patient at the scene, using taxis, and transporting to clinics) vs taking patients directly to an emergency department (ED). Studies show that patients favorably support these alternative options but have not included the perspectives of caregivers of children. Our objective was to describe caregivers’ views about these alternative disposition processes and analyze whether caregiver support is associated with sociodemographic factors.

**Methods:**

We surveyed a convenience sample of caregivers in a pediatric ED. We asked caregivers 15 questions based on a previously validated survey. We then conducted logistic regressions to determine whether sociodemographic factors were associated with levels of support.

**Results:**

We enrolled 241 caregivers. The median age of their children was five years. The majority of respondents were non-Hispanic Black (57%) and had public insurance (65%). We found that a majority of respondents supported all alternative EMS disposition options. The overall level of agreement for survey questions ranged from 51–93%. We grouped questions by theme: non-transport; alternative destinations; communication with EMS physician; communication with primary care physician and sharing records; restricted EMS role; and shared decision-making. Regression analyses for each theme found that race/ethnicity, public insurance, and patient age were not significantly associated with the level of support.

**Conclusion:**

Most caregivers were supportive of alternative EMS disposition options for children with low-acuity complaints. Support did not vary significantly by respondent race/ethnicity, public insurance status, or patient age.

## INTRODUCTION

Emergency medical services (EMS) call volumes have increased to more than 20 million annual EMS responses in the United States^1^ compared to 17 million calls just 10 years ago.^2^ Pediatric transports represent 13% of all EMS transports in the US.^3^ Many of these patients may have low-acuity complaints and not need the medical resources associated with an ambulance transport or emergency department (ED) encounter.^4^,^5^ Studies have found that between 10–60% of all EMS transports might be safely transported to alternative destinations other than the ED,^6^–^8^ but this percentage is unknown for pediatric patients. Enabling children with low-acuity complaints to be transported by other means, or seen in healthcare settings other than the ED, could provide more efficient, cost-effective, and patient-centered care.^9^

Industry experts and federal funding agencies have recommended pilot studies of alternative EMS disposition processes.^10^,^11^ *EMS Agenda 2050* envisages that in the future, “EMS and its partner agencies will coordinate to provide the most appropriate care to the patient, with transport to a healthcare facility being just one option.^”12^ In 2019, the Centers for Medicare & Medicaid Services (CMS) launched the *Emergency Triage, Treatment & Transport (ET3)* model. The *ET3* provides incentives for EMS agencies to develop and assess protocols for Medicare patients so that they may be assessed at the scene (including with the use of telemedicine) and not transported or transported to a primary care office.^13^ Research into more flexible EMS delivery and reimbursement processes is a frequently identified priority area for prehospital pediatric research.^14^,^15^

Successful implementation of alternative EMS disposition processes will require understanding the perspectives of patients and caregivers.^16^,^17^ For example, community engagement and acceptance have been critical in successfully implementing community paramedicine programs.^18^–^20^ A Resource Document for the National Association of EMS Physicians notes that caregiver expectations may preclude including children in alternative disposition programs.^21^ Patients have previously been supportive of alternative EMS dispositions, with approval ratings of 50–90%.^22^,^23^ These studies have included limited numbers of pediatric caregivers and non-White respondents. Therefore, we performed this study to address this gap in the literature and capture the perspectives of caregivers of children. Previous studies have described the specific vulnerabilities of young children,^24^–^27^ different levels of trust in the healthcare system by race/ethnicity^28^–^30^ and disparities in EMS care associated with race^31^–^34^ and economic status.^35^ We therefore hypothesized that caregivers of young children and those from racial-ethnic minorities would have lower levels of support for alternative EMS processes than was previously described in the literature.

## METHODS

### Study Design

We conducted a cross-sectional survey of caregivers presenting to an urban, academic pediatric ED between August 2018–January 2019. This study took place at a freestanding children’s hospital with a Level I pediatric trauma center with an annual volume of approximately 90,000 emergency patient encounters. The hospital receives almost all EMS pediatric transports from the District of Columbia, and the majority of pediatric EMS transports from two neighboring counties in Maryland. Our institutional review board approved this study.

### Data Source and Collection

We used a previously validated survey developed by Munjal et al.^22^ with the addition of questions specific to a 911-linked nurse triage line (Supplemental Figure 1). We asked caregivers their level of agreement with 15 statements on a five-point Likert scale (strongly agree, agree, neutral, disagree, strongly disagree). The survey also asked whether the caregiver had called 911 over the prior three years and whether the patient had arrived by ambulance on the date surveyed. Caregivers were eligible for inclusion regardless of mode of arrival. We approached caregivers in the waiting room or clinical exam room after they had completed initial ED triage. Caregivers were approached consecutively during open enrollment periods when research staff were available (Monday–Friday from 8 am–11 pm, and Saturday–Sunday from 2 pm–10 pm). Research associates (RA) collected the survey responses on an electronic tablet device using REDCap electronic data capture tools hosted at The Clinical and Translational Science Institute (CTSI) at Children’s National (Research Electronic Data Capture).^36^,^37^ The RAs then reviewed the patient record to collect additional data, including patient demographics, triage acuity level, and chief complaint. Caregivers provided demographic information to registration staff. The study enrollment workflow is outlined in Supplemental Figure 2. Only IRB-authorized study team members had access to the password-protected and Health Insurance Portability and Accountability-compliant REDCap platform.

*Population Health Research Capsule*

What do we already know about this issue?

*Adult patients are supportive of alternative EMS dispositions (including leaving at scene, using taxis, and transporting to clinics) for non-emergent calls.*

What was the research question?

*Are caregivers supportive of including children in alternative EMS disposition programs?*

What was the major finding of the study?

*Most caregivers are supportive of including children in alternative EMS disposition programs.*

How does this improve population health?

*Including appropriate children in alternative EMS disposition programs could provide more efficient and patient-centered care.*

### Data Analysis

We decided a priori to collect an initial sample of approximately 250 patients to enable us to perform multivariable modeling with 12 predictor variables for the outcome of caregiver agreement (assuming at least 50% respondent agreement). The primary objective of our study was to describe the overall level of support for specific components of an alternative EMS disposition process. We decided a priori to group “agree” and “strongly agree” responses together. The secondary objective of our study was to determine whether support for components of an alternative EMS disposition process was associated with race/ethnicity or insurance status. We used bivariable regression analyses for each survey question to determine the association with race/ethnicity and insurance status.

We then grouped questions into six themes (non-transport, alternative destinations, communication with EMS clinician, communication with primary care physician and sharing of records, restricted EMS role, and shared decision-making). We repeated the bivariable logistic regression analyses based on respondents who agreed with *all* questions grouped within a theme. We decided a priori to adjust our final multivariable regression models for patient demographic factors, including age, race/ethnicity, gender, insurance status, state of residence, and other patient encounter variables. Other encounter variables included in the regression analysis were as follows: arrival by ambulance on day of survey completion; use of an ambulance in the prior three years; day of week; hour of arrival; and Emergency Severity Index (ESI) triage level on the date of visit. All statistical analysis was conducted using SAS software version 9.3 (SAS Institute Inc., Cary, NC).

## RESULTS

We enrolled 241 caregivers. The median patient age was five years (interquartile range 18 months–10 years), and 56% were male. The most common racial/ethnicity responses were non-Hispanic Black (57%) and Hispanic (26%). Most patients were enrolled in public insurance programs (65%). These sample characteristics are similar to overall ED patient demographics at our institution. Almost one-quarter of caregivers stated they had called 911 in the prior three years, while only 14% of respondents had arrived in the ED by ambulance on the day of survey enrollment (Table 1).

The overall level of agreement for survey questions ranged from 51–93%. For ease of interpretation, we grouped questions into themes that addressed specific components of alternative EMS disposition processes: non-transport; alternative destinations; communication with EMS clinicians; communication with primary care physicians and sharing of medical records; a restricted role for EMS; and shared decision-making. These themes align with those used in previously published literature using this survey.^22^ We found the highest levels of support for caregiver involvement in shared decision-making; 93% of respondents agreed with the statement, “I would prefer to be involved in the decision as to if and where my child is to be transported” (Q12). There were also very high levels of support for the sharing of medical records and information; 89% of caregivers agreed with the statement, “When treated by EMS, the EMS professionals should have access to my child’s medical history in order to treat them correctly” (Q1), and 87% agreed with the statement “I would feel comfortable with EMS sending information about my child’s care electronically to my child’s doctor or hospital’s health records” (Q13). The statements with the lowest level of support pertained to EMS deciding not to transport a patient, with 51% of caregivers agreeing with the statement “I would prefer my child being treated and allowed to stay at home rather than be transported to the hospital if EMS determines they do not need to go to the hospital” (Q4) (Table 2).

Participants were told that a 911-nurse triage line involves a nurse speaking with parents after they have called 911, to determine whether an ambulance is needed. After hearing this brief description, 61% of caregivers agreed with the statement “I would feel comfortable speaking to the nurse triage line operator by telephone and following their advice” (Q15). We found that 63% of caregivers agreed with the statement “I would prefer my child received an urgent appointment at a clinic or primary care doctor’s office rather than being transported to the emergency room if the nurse triage line operator determines that they do not need to go to the hospital” (Q14) (Table 2).

We used White, Non-Hispanic, and private health insurance as our reference group in separate bivariable analyses and did not identify any significant association between those variables and caregiver level of support for any survey question. We ran additional bivariable regression analyses for all other covariates and did not find any variables with a significant association with the level of caregiver support. In our adjusted models, we similarly did not identify any patient or encounter variables associated with support for any specific survey question (Supplemental Table 1) or component theme of an alternative EMS disposition process (Table 3).

## DISCUSSION

A majority of caregivers in this study were supportive of including children in alternative EMS disposition processes. Our results do not support our hypothesis that child age, race/ethnicity, and insurance status would be associated with the level of caregiver support for any aspect of an alternative EMS disposition process. There is currently very little literature regarding caregiver preferences for alternative EMS dispositions for children and no data regarding caregiver attitudes toward a 911-linked nurse triage line. The levels of support for alternative EMS disposition processes in our study are similar to the findings in previous studies with adults.^22^,^23^ Caregiver support for specific statements in our study ranged from 51.0–92.9%. This is very similar to the levels of support (48.2–93.8%) found by Munjal et al when they first developed these survey questions. Furthermore, in both studies the highest levels of support were observed for questions involving shared decision-making and communication and sharing of medical records. In both studies, lowest levels of support were noted for non-transport by EMS.

Previous data from our institution shows significant rates of low-acuity pediatric EMS utilization.^38^ This study suggests that, notwithstanding their current utilization rates, caregivers are supportive of alternative EMS processes of care, irrespective of caregiver race/ethnicity, insurance status, and patient age. Possible explanations for this include that an alternative EMS disposition system provides prompt access to a medical expert to assist with triage, transportation, and prompt access to sick-visit appointments. Qualitative research approaches would help to explore further why our patients do not currently make use of these alternatives despite apparent high levels of support for them.

Even though a higher proportion of pediatric EMS calls are for low-acuity complaints than adult EMS calls, children have been excluded from the vast majority of community paramedicine programs^39^ and other local initiatives that triage specific EMS calls to sites of care other than the ED.^40^–^42^ A recent study found that 19% of all pediatric 911 calls in the US end with a caregiver refusal of transport.^43^ This is substantially higher than the level of patient refusals for adults. Possible reasons for excluding children from EMS-initiated non-transport protocols include the following: children use EMS at lower rates than adults^3^; pilot programs have focused on disease processes more common in adults than children^41^,^44^,^45^; difficulty adapting triage criteria to younger (and sometimes non-verbal) patients; and concern about the acceptability of these alternative processes to caregivers.^46^ Our study, however, suggests that caregiver support for including children in alternative disposition processes is similar to that reported in adults. Only a slight majority of caregivers, however, supported EMS leaving patients at the scene. Successful implementation of alternative disposition processes for children will require the proposed alternatives to be acceptable to the communities that they are designed to serve. Furthermore, studies will also be needed to ensure that any pediatric protocols are safe (with a low rate of under-triage by EMS) and equitable prior to widespread implementation.

## LIMITATIONS

There are several limitations to our study. First, this was a single-center study undertaken in an urban area with most respondents identifying as Black or non-Black Hispanic. These findings should not be applied to other populations. Second, this data was collected before the coronavirus 2019 pandemic. Families and EMS agencies have been eager to reduce unnecessary EMS transports and ED visits during the pandemic,^47^,^48^ which is not captured by our data. Third, the additional questions in our survey relating to a nurse triage line closely mirrored the format of the previously validated survey. We did not, however, separately validate these individual questions.

Additionally, there are specific limitations related to our survey methodology. We may have selection bias, as this was a sample of caregivers in the ED when RAs were available to enroll participants. While our patient sample had similar demographics to overall ED patient data, social factors affecting the use of EMS may be different for children arriving overnight. Low-acuity pediatric EMS calls are more common overnight than during usual office hours.^38^,^49^ This likely reflects lack of other sources of available care overnight. Very few caregivers declined to complete this survey; therefore, we do not believe there is a significant non-response bias. Despite explaining that the research team was not responsible for implementation of alternative EMS disposition protocols, there may be acquiescence bias with caregivers believing the RAs wanted to hear approval of these alternative dispositions. Finally, we asked these questions in the same order, consistent with the previous study that validated the survey. This may have generated question-order bias.

## CONCLUSION

Caregiver support for alternative EMS disposition processes for children is similar to published rates for adult patients. We found high levels of support for most components of an alternative EMS disposition process, although almost half of caregivers were opposed to being left at the scene if EMS determined transport was not necessary. Levels of support did not vary significantly with caregiver insurance status or race/ethnicity. Our study directly refutes the assertion that caregiver expectations should automatically preclude children from being included in alternative EMS disposition programs. Further qualitative studies should explore why caregivers have variable levels of support for the component parts of an alternative EMS disposition process. Caregiver perspectives could also be used to develop specific alternative EMS disposition protocols that are patient centered. These protocols would then need to be prospectively evaluated.

## Supplementary Information







## Figures and Tables

**Table 1:** Selected population characteristics for children of the enrolled caregivers (N = 241).

Characteristic	n (%)
Age category	
Less than 1 y/o	33 (13.7%)
1 y/o to 3 y/o	63 (26.1%)
4 y/o to 6 y/o	51 (21.2%)
7 y/o to 12 y/o	49 (20.3%)
Greater than 12 y/o	45 (18.8%)
Gender	
Female	107 (44.4%)
Male	134 (55.6%)
Race/Ethnicity	
White, Non Hispanic	33 (13.7%)
Black, Non Hispanic	138 (57.3%)
Hispanic	62 (25.7%)
Other	8 (3.3%)
State	
DC	131 (54.4%)
Other	110 (45.6%)
Insurance status	
Private	70 (29.1%)
Public	157 (65.2%)
Not documented	14 (5.8%)
Triage ESI level	
Levels 1 and 2	38 (15.8%)
Level 3	104 (43.2%)
Level 4	85 (35.3%)
Level 5	14 (5.8%)
Arrived via ambulance	33 (13.7%)
Arrived during business hours	118 (49.0%)
Called 911 in the last 3 years	56 (23.2%)

*y/o*, years old; *DC*, District of Columbia; *ESI*, Emergency Severity Index.

**Table 2:** Caregiver levels of agreement to survey items.

Survey questions	% Strongly Agree/Agree
Non-transport	
Q2 Sometime EMS can treat a child and they no longer need to go to the hospital.	56.0%
Q4 I would prefer my child being treated and allowed to stay at home rather than be transported to the hospital if EMS determines they do not need to go to the hospital.	51.0%
Q7 I want EMS to do an evaluation of my child and then advise me whether they need to go to the hospital.	72.9%
Alternative destinations	
Q3 EMS should have the option to bring children to a primary care office, urgent care center or clinic,	73.9%
Q5 I would prefer my child being taken to a clinic or primary care doctor’s office rather than to the emergency room if EMS determines that they do not need to go to the hospital.	57.7%
Q14 I would prefer my child received an urgent appointment at a clinic or primary care doctor’s office rather than being transported to the emergency room if the Nurse Triage Line operator determines that they do not need to go to the hospital.	63.1%
Communication with EMS clinician	
Q8 I would feel comfortable speaking to the EMS supervising doctor by telephone and following their advice.	56.4%
Q9 I would feel comfortable speaking to the EMS supervising doctor by videophone and following their advice.	58.5%
Q15 I would feel comfortable speaking to the Nurse Triage Line operator by telephone and following their advice.	61.0%
Communication with primary care physician and sharing records	
Q1 When treated by EMS, the EMS professionals should have access to my child’s medical history in order to treat them correctly.	89.2%
Q10 I would feel comfortable if EMS communicated with my child’s doctor and together made a decision about my child’s treatment and transport destination.	76.4%
Q11 I would feel comfortable if EMS communicated with my child’s doctor and together decided my child did not need to be transported.	65.6%
Q13 I would feel comfortable with EMS sending information about my child’s care electronically to my child’s doctor or hospital’s health records.	86.7%
Restricted EMS role	
Q6 EMS should not be restricted to only providing lifesaving treatment.	57.7%
Shared decision making	
Q12 I would prefer to be involved in the decision as to if and where my child is to be transported.	92.9%

*EMS*, emergency medical services.

**Table 3:** Factors associated with agreement to all survey items within a theme.

	Non-transport Q2, Q4, Q7 aOR (95% CI)	Alternative destinations Q3, Q5, Q14 aOR (95% CI)	Communication with EMS clinician Q8, Q9, Q15 aOR (95% CI)	Communication with PCP & Sharing Records Q1, Q10, Q11, Q13 aOR (95% CI)	Restricted EMS Role Q6 aOR (95% CI)	Shared Decision Making Q12 aOR (95% CI)
Age category						
Less than 1 y/o	2.7 (1.0, 7.4)	1.6 (0.6, 4.2)	2.7 (1.0, 7.1)	1.0 (0.4, 2.5)	0.4 (0.1, 1.0)	0.4 (0.1, 2.8)
1 y/o to 3 y/o	1.5 (0.6, 3.7)	1.5 (0.6, 3.4)	2.0 (0.8, 4.8)	0.9 (0.4, 2.0)	0.3 (0.1, 0.8)^*^	0.8 (0.2, 3.9)
4 y/o to 6 y/o	2.1 (0.8, 5.2)	1.8 (0.7, 4.2)	2.8 (1.2, 6.8)^*^	1.7 (0.7, 4.3)	0.4 (0.2, 1.0)	1.7 (0.3, 11.1)
7 y/o to 12 y/o	1.7 (0.7, 4.3)	1.2 (0.5, 2.9)	1.9 (0.8, 4.8)	1.5 (0.6, 3.6)	0.3 (0.1, 0.8)^*^	1.2 (0.2, 6.7)
Greater than 12 y/o	Referent	Referent	Referent	Referent	Referent	Referent
Gender						
Female	1.2 (0.7, 2.1)	0.9 (0.5, 1.6)	1.0 (0.6, 1.8)	1.3 (0.7, 2.3)	0.6 (0.3, 0.9)^*^	1.0 (0.3, 2.8)
Male	Referent	Referent	Referent	Referent	Referent	Referent
Race/Ethnicity						
White, Non Hispanic	Referent	Referent	Referent	Referent	Referent	Referent
Black, Non Hispanic	0.6 (0.2, 1.4)	0.7 (0.3, 1.7)	0.8 (0.3, 1.8)	0.5 (0.2, 1.4)	0.8 (0.3, 1.9)	0.8 (0.1, 5.1)
Hispanic	0.8 (0.3, 2.2)	0.8 (0.3, 2.1)	0.6 (0.2, 1.7)	0.8 (0.3, 2.4)	0.4 (0.1, 1.2)	0.9 (0.1, 7.0)
Other	1.7 (0.3, 8.9)	0.3 (0.0, 1.7)	0.9 (0.2, 4.3)	1.0 (0.2, 6.2)	0.2 (0.0, 1.0)	0.6 (0.0, 8.5)
State						
DC	1.1 (0.6, 2.0)	1.2 (0.7, 2.2)	0.9 (0.5, 1.7)	0.8 (0.5, 1.5)	1.2 (0.7, 2.2)	0.9 (0.3, 2.7)
Other	Referent	Referent	Referent	Referent	Referent	Referent
Insurance status						
Private	Referent	Referent	Referent	Referent	Referent	Referent
Public	0.6 (0.3, 1.2)	0.9 (0.4, 1.8)	0.9 (0.4, 1.7)	0.8 (0.4, 1.8)	1.1 (0.5, 2.2)	0.7 (0.2, 2.6)
Not documented	0.9 (0.3, 3.2)	1.0 (0.3, 3.6)	0.9 (0.2, 2.8)	0.2 (0.1, 0.9)	0.6 (0.2, 2.0)	--
ESI triage level						
Levels 1 and 2	1.9 (0.8, 4.4)	0.9 (0.5, 2.0)	2.0 (0.9, 4.4)	1.3 (0.6, 3.0)	1.9 (0.8, 4.6)	0.5 (0.1, 2.2)
Level 3	1.9 (1.0, 3.5)	1.0 (0.5, 1.8)	1.2 (0.7, 2.2)	1.0 (0.5, 1.8)	1.3 (0.7, 2.3)	0.8 (0.2, 2.7)
Levels 4 and 5	Referent	Referent	Referent	Referent	Referent	Referent
Arrived via ambulance	1.0 (0.4, 2.2)	1.5 (0.7, 3.3)	1.0 (0.5, 2.3)	1.1 (0.5, 2.5)	0.6 (0.2, 1.3)	0.3 (0.1, 0.9)^*^
Arrived in business hours	1.4 (0.8, 2.4)	1.4 (0.8, 2.4)	1.4 (0.8, 2.4)	1.4 (0.8, 2.5)	0.8 (0.5, 1.4)	1.4 (0.5, 4.2)
Called 911 in the last 3 years	0.6 (0.3, 1.2)	0.5 (0.2, 1.0)	0.8 (0.4, 1.5)	0.5 (0.2, 0.9)^*^	0.7 (0.4, 1.4)	3.5 (0.7, 17.4)

*a/OR*, adjusted odds ratio; *CI*, confidence interval; *DC*, District of Columbia; *y/o*, years old; *ESI*, Emergency Severity Index.
