# Supplementary material for: Caregiver Perceptions Regarding Alternative Emergency Medical Services Dispositions for Children: A Cross-Sectional Survey Analysis
Source: West J Emerg Med. 2022 Jul 2;23(4):489–96. doi: 10.5811/westjem.2022.5.55470 (PMC9391016; doi:10.5811/westjem.2022.5.55470)
Supplement: Supplementary file 1 [file wjem-23-489-s001.pdf]

EMS Survey

Please complete the survey below.

1) Did your child arrive by ambulance today?
{[q1] yesno}

◯ Yes
◯ No

2) Have you called 911 for this child (or another child in your care) in the last 3 years?
{[q2] yesno}

◯ Yes
◯ No

3) When treated by EMS, the EMS professionals should have access to my child's medical history in order to treat them correctly
{[q3] radio}

◯ {1} Strongly Agree ◯ {2} Agree ◯ {3} Neutral ◯ {4} Disagree ◯ {5} Strongly Disagree

4) Sometime EMS can treat a child and they no longer need to go to the hospital
{[q4] radio}

◯ {1} Strongly Agree ◯ {2} Agree ◯ {3} Neutral ◯ {4} Disagree ◯ {5} Strongly Disagree

5) EMS should have the option to bring children to a primary care office, urgent care center or clinic
{[q5] radio}

◯ {1} Strongly Agree ◯ {2} Agree ◯ {3} Neutral ◯ {4} Disagree ◯ {5} Strongly Disagree

6) I would prefer my child being treated and allowed to stay at home rather than be transported to the hospital if EMS determines they do not need to go to the hospital
{[q6] radio}

◯ {1} Strongly Agree ◯ {2} Agree ◯ {3} Neutral ◯ {4} Disagree ◯ {5} Strongly Disagree

7) I would prefer my child being taken to a clinic or primary care doctor's office rather than to the emergency room if EMS determines that they do not need to go to the hospital
{[q7] radio}

◯ {1} Strongly Agree
◯ {2} Agree
◯ {3} Neutral
◯ {4} Disagree
◯ {5} Strongly Disagree

8) EMS should be restricted to only providing lifesaving treatment
{[q8] radio}

◯ {1} Strongly Agree ◯ {2} Agree ◯ {3} Neutral ◯ {4} Disagree ◯ {5} Strongly Disagree

9) I want EMS to do an evaluation of my child and then advise me whether they need to go to the hospital
{[q9] radio}

◯ {1} Strongly Agree ◯ {2} Agree ◯ {3} Neutral ◯ {4} Disagree ◯ {5} Strongly Disagree

10) I would feel comfortable speaking to the EMS supervising Doctor by telephone and following their advice
{[q10] radio}

◯ {1} Strongly Agree ◯ {2} Agree ◯ {3} Neutral ◯ {4} Disagree ◯ {5} Strongly Disagree

11) I would feel comfortable speaking to the EMS supervising Doctor by videophone and following their advice
{[q11] radio}

◯ {1} Strongly Agree ◯ {2} Agree ◯ {3} Neutral ◯ {4} Disagree ◯ {5} Strongly Disagree

12) I would feel comfortable if EMS communicated with my child's doctor and together made a decision about my child's treatment and transport destination
{[q12] radio}

◯ {1} Strongly Agree ◯ {2} Agree ◯ {3} Neutral ◯ {4} Disagree ◯ {5} Strongly Disagree

13) I would feel comfortable if EMS communicated with my child's doctor and together decided my child did not need to be transported
{[q13] radio}

◯ {1} Strongly Agree ◯ {2} Agree ◯ {3} Neutral ◯ {4} Disagree ◯ {5} Strongly Disagree

14) I would prefer to be involved in the decision as to if and where my child is to be transported
{[q14] radio}

◯ {1} Strongly Agree ◯ {2} Agree ◯ {3} Neutral ◯ {4} Disagree ◯ {5} Strongly Disagree

15) I would feel comfortable with EMS sending information about my child's care electronically to my child's doctor or hospital's health records
{[q15] radio}

◯ {1} Strongly Agree ◯ {2} Agree ◯ {3} Neutral ◯ {4} Disagree ◯ {5} Strongly Disagree

Some regions in the country have started to have use Nurse Triage Lines. This means that when you call 911, some less critical calls may be transferred to a Nurse who would provide advice. After asking some questions, they may then arrange for a same day appointment at a primary care office or urgent care clinic. They may also help arrange for transport to that appointment if necessary.

16) I would prefer my child received an urgent appointment at a clinic or primary care doctor's office rather than being transported to the emergency room if the Nurse Triage Line operator determines that they do not need to go to the hospital
{[q16] radio}

◯ {1} Strongly Agree ◯ {2} Agree ◯ {3} Neutral ◯ {4} Disagree ◯ {5} Strongly Disagree

17) I would feel comfortable speaking to the Nurse Triage Line operator by telephone and following their advice
{[q17] radio}

◯ {1} Strongly Agree ◯ {2} Agree ◯ {3} Neutral ◯ {4} Disagree ◯ {5} Strongly Disagree
