# Supplementary material for: Caregiver Perceptions Regarding Alternative Emergency Medical Services Dispositions for Children: A Cross-Sectional Survey Analysis
Source: West J Emerg Med. 2022 Jul 2;23(4):489–96. doi: 10.5811/westjem.2022.5.55470 (PMC9391016; doi:10.5811/westjem.2022.5.55470)
Supplement: Supplementary file 3 [file wjem-23-489-s003.docx]

**Supplemental Figure 2: Workflow for enrolling subjects**
